# Supplementary material for: Interactions between Filter-Feeding Bivalves and Toxic Diatoms: Influence on the Feeding Behavior of Crassostrea gigas and Pecten maximus and on Toxin Production by Pseudo-nitzschia
Source: Toxins (Basel). 2021 Aug 19;13(8):577. doi: 10.3390/toxins13080577 (PMC8402372; doi:10.3390/toxins13080577)
Supplement: Supplementary file 1 [file toxins-13-00577-s001.zip › toxins-1277017 supplementary update.pdf]

Supplementary Materials: Interactions between Filter-Feeding Bivalves and Toxic Diatoms: Influence on the Feeding Behavior of *Crassostrea gigas* and *Pecten maximus* and on Toxin Production by *Pseudo-nitzschia*

Aurore Sauvey, Françoise Denis, Hélène Hégaret, Bertrand Le Roy, Christophe Lelong, Orianne Jolly, Marie Pavie and Juliette Fauchot

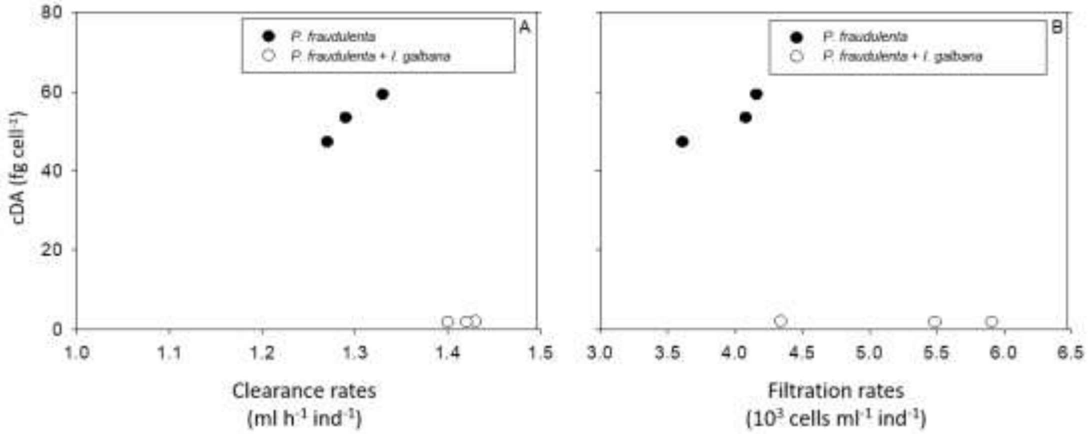

Figure S1. cDA concentrations in *P. fraudulenta* (fg cell^{-1}) as a function of the average clearance rates (mL h^{-1} ind^{-1}, A) and the average filtration rates (cells mL^{-1} ind^{-1}, B) of *C. gigas* over the 5 days of the experiment.

Table S1. Concentrations of dissolved domoic acid (dDA, pg mL^{-1}) in the media containing *P. australis* or *P. fraudulenta* at the beginning and the end of the exposure experiments. * significant difference with a p-value of 0.05.

		dDA (pg ml^{-1})	
		P. fraudulenta	*P. australis*
with *C. gigas*	Initial	0.04 ± 0.03	1.58 ± 0.29
	Pseudo-nitzschia with T. galbana	0.06 ± 0.02	1.59 ± 0.54
	Pseudo-nitzschia alone	0.05 ± 0.02	1.62 ± 0.24
	Control	0.05 ± 0.03	1.46 ± 0.27
with *P. maximus*	Initial	0.28 ± 0.08	2.15 ± 0.24
	Pseudo-nitzschia with T. galbana	0.36 ± 0.13	4.13 ± 0.90 *
	Control	0.25 ± 0.02	2.33 ± 0.07

Table S2. Nutrient concentrations (µmol L^{-1}) in the culture medium in the control, at the beginning and the end of the *C. gigas* and *P. maximus* exposure experiments.

	Conditions	Time (h)		Nutrient concentrations (µmol L^{-1}) NO_3^-	PO_4^{3-}	$Si(OH)_4$
Experiments with *C. gigas*	Condition 1 *C. gigas* + *P. fraudulenta* + *T. galbana*	Initial	0 h	306 ± 65	22 ± 1	61 ± 5
		Final	96 h	304 ± 35	14 ± 1	34 ± 3
		Control	96 h	343 ± 9	18 ± 2	49 ± 7
	Condition 2 *C. gigas* + *P. australis* + *T. galbana*	Initial	0 h	559 ± 23	15 ± 2	84 ± 42
		Final	120 h	475 ± 74	14 ± 1	69 ± 13
		Control	120 h	294 ± 122	8 ± 1	52 ± 2
	Condition 3 *C. gigas* + *P. fraudulenta*	Initial	0 h	311 ± 12	16 ± 0.5	57 ± 8
		Final	96 h	305 ± 20	15 ± 1.5	32 ± 4
		Control	96 h	302 ± 22	11 ± 0.5	37 ± 3
	Condition 4 *C. gigas* + *P. australis*	Initial	0 h	305 ± 2	16 ± 0.5	57 ± 18
		Final	120 h	237 ± 11	12 ± 0.5	54 ± 3
		Control	120 h	360 ± 31	11 ± 1	36 ± 3
Experiments with *P. maximus*	Condition 1 *P. maximus* + *P. fraudulenta* + *T. galbana*	Initial	0 h	140 ± 16	13 ± 1	48 ± 3
		Final	6 h	117 ± 22	13 ± 3	41 ± 19
		Control	6 h	197 ± 13	14 ± 1	34 ± 7
	Condition 2 *P. maximus* + *P. australis* + *T. galbana*	Initial	0 h	304 ± 103	11 ± 1	86 ± 15
		Final	6 h	230 ± 152	7 ± 1	33 ± 3
		Control	6 h	229 ± 39	13 ± 2	35 ± 10
